# Nickel accumulation in leaves, floral organs and rewards varies by serpentine soil affinity

**DOI:** 10.1093/aobpla/plu036

**Published:** 2014-06-30

**Authors:** George A. Meindl, Daniel J. Bain, Tia-Lynn Ashman

**Affiliations:** 1 Department of Biological Sciences, University of Pittsburgh, Pittsburgh, PA 15260, USA; 2 Department of Geology and Planetary Science, University of Pittsburgh, Pittsburgh, PA 15260, USA

**Keywords:** Brassicaceae, endemism, flowers, metal accumulation, nickel, serpentine soil.

## Abstract

Serpentine soils are edaphically stressful environments that host many endemic plant species. In particular, serpentine soils are high in several heavy metals (e.g. nickel, cobalt and chromium) and these high heavy metal concentrations are thought, in part, to lead to varying levels of plant adaptation and soil affinities (i.e. endemic vs. non-endemic plant species). It is unclear, however, whether serpentine endemics vs. non-endemics differ with respect to heavy metal uptake into either vegetative or reproductive organs. Here, we use nickel as a model to determine whether plant heavy metal uptake varies with the level of endemism in several non-hyperaccumulating species. Under controlled greenhouse conditions, we grew seven plant species from the Brassicaceae family that vary in their degrees of affinity to serpentine soil from low (indifferent) to medium (indicator) and high (endemic) in soil that was nickel supplemented or not. We quantified nickel concentrations in leaves, pistils, anthers, pollen and nectar. While nickel concentrations did not vary across organs or affinities when grown in control soils, under conditions of nickel supplementation endemic species had the lowest tissue concentrations of nickel, particularly when considering leaves and pistils, compared with indifferent/indicator species. Species indifferent to serpentines incorporated higher concentrations of nickel into reproductive organs relative to leaves, but this was not the case for indicator species and endemics where nickel concentration was similar in these organs. Our findings suggest that endemic species possess the ability to limit nickel uptake into above-ground tissues, particularly in reproductive organs where it may interfere with survival and reproduction. Indifferent species accumulated significantly more nickel into reproductive organs compared with leaves, which may limit their reproductive potential relative to endemic species when growing on serpentine soils. Additional work determining the fitness consequences of these differences will further our understanding of edaphic endemism.

## Abstract (precis)

In this study, we determined whether nickel uptake varies with level of serpentine endemism and quantified nickel concentrations in leaves, pistils, anthers, pollen and nectar in several plant species. Endemic species had the lowest tissue concentrations of nickel. Species indifferent to serpentines incorporated higher concentrations of nickel into reproductive organs relative to leaves, but this was not the case for indicator species and endemics where nickel was similar in these organs. Our findings suggest that endemic species possess the ability to limit nickel uptake into above-ground tissues, particularly in reproductive organs where it may interfere with survival and reproduction.

## Introduction

Edaphic factors, such as soil texture, depth and chemical composition, are a primary force in shaping the distributions of plant species (Silvertown 2004; Toledo *et al.* 2012; Dubuis *et al.* 2013). While many plant species can be found growing in a variety of habitats, some species become entirely restricted to a particular soil type (i.e. edaphic endemics; Macnair and Gardner 1998; Rajakaruna 2004). Serpentine soils, which can be found on every continent (Brooks 1987), provide one of the most remarkable examples of plant adaptation to atypical soils (O'Dell and Rajakaruna 2011) and geographic regions containing serpentine soil often harbour numerous endemic species (Brooks 1987; Safford *et al.* 2005; Anacker 2011). Serpentine-derived soils represent a nutritionally stressful growing environment for most plants because of a low Ca : Mg ratio, deficiency of essential nutrients (e.g. N, P, K) and high levels of potentially phytotoxic heavy metals (e.g. nickel [Ni], cobalt, chromium; Brooks 1987; Brady *et al.* 2005; Kazakou *et al.* 2008). While comparisons of plant tissue chemistry between endemic and non-endemic species can provide insight into the physiological features of edaphic endemics (Palacio *et al.* 2007), it is unclear whether soil affinity (i.e. endemic vs. non-endemic) affects plant tissue chemistry for serpentine plant species. For example, if endemic species are specifically adapted to the abiotic stresses of serpentine soil, then they might be better able to acquire limiting resources and/or exclude phytotoxic elements from the soil than non-endemic species. Considering that species span a gradient of affinity to serpentine soils, with some only occasionally found on serpentines (i.e. ‘indifferent’, ≤45 % occurrences on serpentines), some commonly found either on or off serpentines (i.e. ‘indicator’, ∼55–64 % occurrences on serpentines) and others entirely restricted to serpentines (i.e. ‘endemic’, ≥95 % occurrences on serpentines) (Safford *et al.* 2005), serpentine soils provide an ideal system to test whether soil affinity affects tissue chemistry.

The elevated heavy metal concentrations, in particular, in serpentine soils are thought to drive levels of plant adaptation (Lazarus *et al.* 2011), as non-adapted species lack physiological mechanisms to avoid metal toxicity (e.g. via metal exclusion or sequestration with chelating agents; Kazakou *et al.* 2008). However, of the few studies examining the differences in metal accumulation between endemic and non-endemic species, some have found decreased shoot metal accumulation in endemic species (Nagy and Proctor 1997; Burrell *et al.* 2012) while others have found no such difference (Fiedler 1985; Lee and Reeves 1989). Plant response to the heavy metal Ni found in serpentines is relatively well studied (Kazakou *et al.* 2008), and provides an excellent model to test whether endemic vs. non-endemic species vary in heavy metal accumulation. While some plants require Ni in trace quantities as an active component of the enzyme urease (Welch 1981), it is generally considered toxic to plants and is implicated in causing abnormal vegetative growth, necrosis and chlorosis of leaves and inhibiting photosynthesis (reviewed in Yusuf *et al.* 2011). Furthermore, Ni is known to negatively impact aspects of plant reproduction for non-hyperaccumulators, such as decreasing pollen germination (Tuna *et al.* 2002; Breygina *et al.* 2012) and seed production (Malan and Farrant 1998) when plants not adapted to elevated Ni conditions are grown in them. However, whether Ni is accumulated into reproductive organs, such as anthers and pistils, has only been studied in one serpentine plant species that is a known Ni hyperaccumulator (i.e. accumulates >1000 ppm Ni [*Streptanthus polygaloides*]; Meindl and Ashman 2014; Sánchez-Mata *et al.* 2014). While Ni hyperaccumulators are often specialized to serpentines (Reeves and Baker 2000), they represent an extreme minority, in both number of taxa and plant–soil interactions, of serpentine endemic plant species (Reeves 2006; Anacker 2011). Beyond these rare, yet relatively well-studied hyperaccumulators (Reeves 2006; Gall and Rajakaruna 2013), it is largely unknown whether the vast majority of serpentine species exhibit significant variation in Ni accumulation into above-ground organs. Therefore, studies are needed that focus on metal uptake for non-hyperaccumulating species to determine more general patterns of metal uptake or exclusion across serpentine plant species. Furthermore, it is unknown whether most serpentine plants accumulate Ni into pollen grains, despite evidence that plants growing in soils contaminated by metals via human activities can accumulate them into pollen (Moroń *et al.* 2012). Metals in pollen could reduce germination (citations above; Mohsenzadeh *et al.* 2011; Yousefi *et al.* 2011*a*) or pollinator attraction (Meindl and Ashman 2014), and Ni accumulation in nectar can affect pollinator foraging (Meindl and Ashman 2013, 2014). Thus, a first and necessary step towards understanding the reproductive consequences of growth on serpentine soil is documenting metal concentrations of reproductive organs and floral rewards of non-hyperaccumulating serpentine plants, as well as determining whether or not non-hyperaccumulating endemic species are better able to avoid potentially deleterious effects of metals by excluding them from reproductive organs than non-endemics. However, explicit experimental comparisons of metal accumulation across a range of species that vary in serpentine affinity, as well as across a range of vegetative and reproductive organs, are lacking.

To test these ideas, we grew seven species of plants from the Brassicaceae family that varied in serpentine soil affinity, but are not considered metal hyperaccumulators, in either control soils or soils supplemented with Ni to determine whether serpentine endemic and non-endemic plants differ with respect to Ni uptake (i.e. accumulate lower or similar concentrations of Ni into leaves, reproductive organs and rewards compared with non-endemic species, respectively). Using Ni as a model for plant response to serpentine heavy metals in general, we answered these questions: (i) Do serpentine endemic and non-endemic species differ in terms of Ni uptake into (a) leaves, (b) pistils, (c) anthers, and/or (d) nectar? (ii) Do serpentine endemics and non-endemics differ in the relative concentrations of Ni in vegetative vs. reproductive organs? (iii) Is Ni incorporated into pollen grains in any of these species?

## Methods

### Study system

Plants in the Brassicaceae family (mustard family) are well represented on California serpentine soils (Safford *et al.* 2005), including the seven species used here that differ in serpentine affinity from strictly endemic (i.e. ≥95 % occurrence on serpentine) to indifferent to serpentine soils (i.e. ≤45 % occurrence on serpentine soils): endemic: *Streptanthus morrisonii*, *S. breweri* var. *breweri*; indicator: *S. glandulosus* ssp. *glandulosu*s, *S. tortuosus*; indifferent: *Hirschfeldia incana*, *Erysimum capitatum* var. *capitatum*, *Boechera breweri* (Table 1). To assign serpentine affinity scores to taxa, we follow the nomenclature used in Safford *et al.* (2005); however, in this work we follow the recent revised nomenclature for two taxa (i.e. *S. tortuosus* var. *suffrutescens* [now *S. tortuosus*] and *Arabis breweri* [now *Boechera breweri*]; Baldwin *et al.* 2012). All are spring flowering, insect pollinated herbaceous annuals or perennials that occur in North America, with four taxa being restricted to California (Table 1). Seeds from each taxon were bulk-collected from a single population per species in the summer of 2012.

**Table 1.** Species descriptions and seed collection locations for all plant species studied. Plants were divided into three categories: serpentine endemic, serpentine indicator or serpentine indifferent. Serpentine affinity score is provided for all taxa, as defined by Safford *et al.* (2005)—species not discussed by Safford *et al.* (2005) are given a score of ‘<1’. Life history (annual or perennial) and distribution ranges are provided for all species (CA, California; NA, North America; AE, Afroeurasia).

Species	Plant category	Habitat affinity score	Life history	Range	Seed collection location
*S. breweri* var. *breweri*	Endemic	5.7	Annual	CA	N38°51′52.4″; W122°24′16.4″
*S. morrisonii*	Endemic	6.1	Perennial	CA	N38°48′45.3″; W122°22′54.9″
*S. glandulosus* ssp. *glandulosus*	Indicator	1.9	Annual	CA	N38°51′43.9″; W122°23′57.3″
*S. tortuosus*	Indicator	1.7	Perennial	Western NA	N39°59′18.4″; W121°17′19.8″
*E. capitatum* var. *capitatum*	Indifferent	<1	Perennial	NA	N41°16′32.5″; W122°41′54.4″
*H. incana*	Indifferent	<1	Annual	NA, AE	N38°51′30.0″; W122°24′35.2″
*B. breweri*	Indifferent	<1	Perennial	CA	N39°57′12.3″: W121°19′4.5″

### Experimental design

Twenty plants per species (total *N* = 140) were grown at the University of Pittsburgh in the fall of 2012. Seeds were subjected to a 4 °C cold and dark treatment for 2 weeks prior to planting. Two weeks after germination, seedlings were transplanted to 27 cm^3^ ‘rocket’ pots (Deepots; Stuewe and Sons, Inc., Tangent, OR, USA) filled with standard potting soil (Fafard #4; Sun Gro Horticulture, Agawam, MA, USA) and six Nutricote^®^ NPK 13-13-13 time-release fertilizer pellets (Arysta LifeScience Corporation, New York, NY, USA). One month after transplanting, all perennials (*S. morrisonii*, *E. capitatum* var. *capitatum*, *S. tortuosus*, *B. breweri*) received a 4 °C cold treatment for 1 month at 8D : 16N. Subsequently, these perennials and the annuals (*S. breweri* var. *breweri*, *H. incana*, *S. glandulosus* ssp. *glandulosus*) were grown under controlled conditions of 12D : 12N, between 70 and 80 °F, until flowering.

One month after potting (annuals), or 1 week after cold treatment (perennials), soil treatment solutions were applied to each plant weekly: either (i) Ni-supplemented (40 mL of 400 ppm Ni nitrate (Ni(NO_3_)_2_-6H_2_O) solution) or (ii) control (40 mL of ammonium nitrate (NH_4_NO_3_) solution to compensate for 190 ppm nitrogen applied to plants in the Ni-supplemented soil treatment). This reflects a natural level of Ni, as serpentine soils contain bioavailable fractions of Ni ranging from 50 to 500 ppm (e.g. L'Huillier and Edighoffer 1996; Chardot *et al.* 2005). Soil treatments were conducted for 4–18 weeks, depending on time to flower. All plants were watered as needed.

### Organ/reward collection and chemical analysis

Three organs (leaves, pistils, anthers) and two floral rewards (pollen, nectar) were collected from individual plants. A single fully developed leaf from the basal rosette was collected from each plant after four soil treatment applications. Pistils, anthers and nectar were collected from the first 5–15 flowers produced per plant. To collect nectar from several flowers per plant we folded a circular piece of filter paper (Whatman^®^ Grade 1; GE Healthcare Bio-Sciences, Pittsburgh, PA, USA) in half and touched it to the floral nectaries in a circular pattern. Nectar volume was determined via the Baker's (1979) spot-staining method, as described in Kearns and Inouye (1993). The measured diameter (mm) of each nectar spot was compared with a standard table that relates spot diameter to nectar volume (μL). This technique is valid for nectars with sugar concentrations ranging from 10 to 50 % and nectar spot diameters ≤12 mm, which is true for many Brassicaceae members (e.g. Masierowska 2003; Nedić *et al.* 2013) including those in our study. Pistils and anthers were dissected from freshly opened whole flowers using forceps. While leaves were collected from every plant (*N* = 10 per species-soil treatment), some plants (*N* = 5) never flowered and thus 7–10 plants were sampled per species-soil treatment for floral organs and rewards. In addition, pollen was collected from two plants per species-soil treatment from an independent set of mature anthers.

Prior to chemical analysis, leaves and pistils were rinsed with diH_2_O and dried at 60 °C for 48 h. Anther, pollen and nectar samples were allowed to air dry for 48 h in microcentrifuge tubes. Samples were weighed to the nearest 0.0001 g on a AE200 Mettler^®^ analytical balance (Mettler-Toledo; LLC, Columbus, OH, USA) and microwave digested in 2–4 mL of trace metal grade HNO_3_ and brought to a final volume of 12–14 mL with MilliQ (Millipore, Bedford, MA, USA) H_2_O. Concentration of Ni is reported as ppm in organs and pollen (i.e. mg kg^−1^) and nectar (i.e. μL L^−1^) and was determined via inductively coupled plasma mass spectrometry (ICP-MS, NEXION 300X; PerkinElmer, Waltham, MA, USA) at the University of Pittsburgh (for details see Meindl and Ashman 2014).

### Statistical analysis

All statistical analyses were conducted in SAS (2011; version 9.3; SAS Institute Inc., Cary, NC, USA). To evaluate the effect of soil treatment, serpentine habitat affinity and organ/reward type on plant Ni concentration, mixed-model ANCOVA was conducted (PROC MIXED). The model included the fixed effects of soil treatment (Ni supplement, control), serpentine habitat affinity (endemic, indicator or indifferent), organ/reward type (leaves, pistils, anthers or nectar) and their interactions and random factors of individual and species, where species was nested within serpentine habitat affinity (Table 1). The number of Ni applications to the soil was included as a covariate (‘application number’). Denominator degrees of freedom for *F*-tests were determined using the Kenward–Roger approximation, which is preferred for small sample sizes and unbalanced data (Littell *et al.* 2002). For Ni-treated plants only, we used pre-planned contrasts to determine whether endemic species (i) incorporated less Ni than indicator/indifferent species in organs/rewards, and (ii) displayed lower concentrations of Ni in reproductive organs (anthers, pistils) relative to leaves than indicator/indifferent species using the CONTRAST option. We used a Student's *t*-test (PROC TTEST) to determine if pollen Ni concentration was higher in Ni-treated plants than in controls. For all analyses, Ni concentrations were natural-log transformed to improve normality of residuals. Back-transformed lsmeans (and 95 % confidence intervals) of Ni concentrations are presented for clarity.

## Results

**Figure 1.**
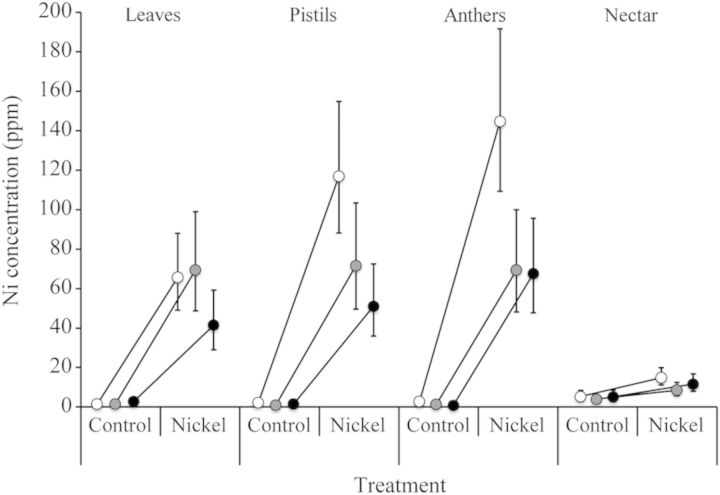
Nickel concentrations among serpentine indifferent (*H. incana*, *E. capitatum* var. *capitatum*, *B. breweri*), indicator (*S. tortuosus*, *S. glandulosus* ssp. *glandulosus*) and endemic (*S. morrisonii*, *S. breweri* var. *breweri*) plant species when grown in control vs. nickel-supplemented soils by organ/reward type (vegetative organ [leaves], two reproductive organs [pistils, anthers] and one floral reward [nectar]). Symbols represent back-transformed lsmeans (±95 % CI): white symbols, indifferent species; grey symbols, indicator species; black symbols, endemic species.

Soil Ni treatment was effective, as mean Ni concentrations in Ni-treated plants were 16 times higher than control plants across all organs and nectar (46.5 vs. 3.00 ppm), but the effect of habitat affinity on Ni in plant tissue was dependent on both soil treatment and organ/reward type (habitat affinity × soil treatment × organ/reward type: *P* < 0.05; Table 2; Fig. 1). Within the Ni soil treatment, endemic species had lower Ni concentrations in leaves and pistils compared with both indicator and indifferent species (leaves—endemic: 39.0 ppm; indicator and indifferent: 62.2 ppm; pistils—endemic: 55.3 ppm; indicator and indifferent: 99.5 ppm; Table 2). However, endemic species did not have significantly lower Ni concentrations compared with indicator/indifferent species in either anthers (endemic: 72.9 ppm; indicator/indifferent: 99.5 ppm; Table 2) or nectar (endemic: 13.1 ppm; indicator/indifferent: 12.8 ppm; Table 2). Furthermore, within the Ni soil treatment, indifferent species had Ni concentrations in anthers/pistils (126.5 ppm) that were twice as high as that in leaves (62.8 ppm; *P* < 0.0001, Table 2). Conversely, we did not detect a difference between endemic and indicator species with respect to concentrations of Ni in reproductive organs relative to vegetative organs (anthers/pistils vs. leaves: endemic 59.7 vs. 39.0 ppm; indicator: 72.2 vs. 66.7 ppm; Table 2).

**Table 2.** Results from mixed-model ANCOVA and pre-planned contrasts of Ni accumulation to leaves, pistils, anthers and nectar (‘organ/reward type’) of seven mustard species that vary in their affinity to serpentine soil (‘habitat affinity’) when grown in either Ni-supplemented or control soils (‘soil treatment’). The number of soil treatment applications (‘application number’) was included as a covariate. Random effects of individual plant (‘individual’) and species (nested within habitat affinity; ‘species (habitat affinity)’ were also included in the model. Significance of fixed effects denoted as *P ≤ 0.05, **P ≤ 0.01 and ***P ≤ 0.0001.

Source of variation	df (num., den.)	*F*
Habitat affinity	2, 1.86	10.42
Soil treatment	1, 119	1204.29***
Organ/reward type	3, 225	8.88***
Application number	1, 13.3	26.57**
Habitat affinity × soil treatment	2, 117	2.18
Habitat affinity × organ/reward type	6, 382	5.16***
Soil treatment × organ/reward type	3, 383	163.22***
Habitat affinity × soil treatment × organ/reward type	6, 383	2.24*

Random effects		*Z*
Individual		3.28**
Species (habitat affinity)		0.19
Pre-planned contrasts		
Endemic vs. non-endemic (indifferent/indicator)		
Leaves	1, 31.5	5.85*
Pistils	1, 32.6	8.24**
Anthers	1, 32.5	3.78
Nectar	1, 32.5	0.02
Vegetative (leaves) vs. reproductive (anthers/pistils)		
Endemic	1, 349	3.15
Indicator	1, 321	0.01
Indifferent	1, 384	20.42***

Across all species, mean Ni concentrations in pollen were 10 times higher in Ni-treated plants than in controls (50.9 vs. 5.7 ppm; *t* = −5.33; df = 26; *P* < 0.0001). Nickel concentration of pollen from Ni-treated plants was highest for indicator species (59.1 ppm), followed by endemic (44.7 ppm) and indifferent (38.9 ppm) species.

## Discussion

Serpentine endemic species incorporated Ni in lower concentrations in leaves and reproductive organs compared with indicator/indifferent species, suggesting that these species may be better adapted to the Ni-rich serpentine soil environment than the indicator/indifferent species studied. However, the magnitude of this difference depended on organ type, as endemics incorporated significantly less Ni into leaves and pistils, but not anthers, compared with indicator/indifferent species. Furthermore, while Ni exclusion is one possible mechanism to limit toxicity, effective sequestration in leaves is another (Yusuf *et al.* 2011). Species indifferent to serpentine had higher Ni concentrations in reproductive organs relative to leaves, whereas endemic and indicator species had similar Ni concentrations across all organs, again suggesting that plant species not regularly associated with serpentines do not possess mechanisms to limit uptake of Ni into reproductive organs.

In the present study, serpentine endemic Brassicaceae exhibited the greatest degree of Ni exclusion, particularly in the leaves and pistils. Similarly, DeHart *et al.* (in review) found that while there was no difference in Ni concentrations between field-collected leaves, flowers or seeds of serpentine endemic and non-endemic species from the plant families Fabaceae, Phrymaceae and Ranunculaceae (possibly due to low levels of phytoavailable Ni in soils at their study sites), endemic species had significantly lower concentrations of the heavy metal cobalt across all organs than non-endemics (DeHart *et al.*, in review). Furthermore, studies of edaphic endemics in other soil environments also suggest that endemic plants may be specialized to their respective soil environment relative to non-endemics. By comparing leaf tissue chemistry of plant species that were either endemic or non-endemic to gypsum soils, which are high in sulfate ions and low in several macronutrients, Palacio *et al.* (2007) concluded that many gypsum soil endemic plants were more efficient at extracting limiting nutrients (e.g. N, P) from gypsum soils relative to non-endemic species, though range size also played a role in the level of specialization observed. Taken together, these findings suggest that many edaphic endemics may be physiologically better suited to their respective soil environments, e.g. by excluding heavy metals or acquiring limiting nutrients, than non-endemics. Our results contribute to ideas that support the specialist model of edaphic endemism (Meyer 1986; Palacio *et al.* 2007) rather than the refuge model, in which endemics are not specifically adapted to a particular soil type (Gankin and Major 1964), although studies of tissue chemistry would have to be coupled with measures of fitness to confirm this idea. It is important to recognize, however, that we intentionally excluded from our study serpentine soil endemics that are known to hyperaccumulate Ni, such as *S. polygaloides*. These species represent an exception to plant metal accumulation by serpentine soil endemics, rather than the rule (Reeves 2006; Anacker 2011), and thus predictions relating to heavy metal accumulation would clearly differ when considering metal hyperaccumulating taxa. However, because metal hyperaccumulation may impart chemical defences to plants (Rascio and Navari-Izzo 2011) and thus may impact plant fitness, documenting metal hyperaccumulation into reproductive organs and rewards in these species may provide valuable insight into the potential adaptive value of metal hyperaccumulation (Boyd and Martens 1992) and warrants additional study. In fact, recent experimental evidence suggests that two Ni hyperaccumulating taxa concentrate Ni in both vegetative and reproductive organs (*S. polygaloides* and *Noccaea fendleri*; Meindl *et al.*, in press). Furthermore, other chemical aspects of serpentine soils besides high Ni concentrations, such as low Ca : Mg ratios, may be equally important drivers of plant adaptation to the serpentine soil environment (Brady *et al.* 2005; O'Dell and Rajakaruna 2011; DeHart *et al.*, in review). Further comparisons of both macronutrient (e.g. Ca, Mg, N, K, P) and heavy metal (e.g. Ni, Co, Cr) concentrations between tissues of endemic and non-endemic species will provide a more comprehensive view of plant adaptation to serpentine soils.

Interestingly, Ni accumulation was, on average, higher in reproductive organs compared with leaves across all species in this study, corroborating similar findings of increased metal accumulation in flowers relative to leaves (Severne 1974; Gabbrielli *et al.* 1997). However, our work suggests that plants restricted to soils with elevated metals generally have lower metal concentrations in floral organs relative to plants not restricted to such soils. This pattern suggests a cost to floral metal accumulation, which could relate to decreased reproductive success via negative impact on pollen and ovule viability, as well as seed and fruit production (Maestri *et al.* 2010). Indeed, recent studies suggest that floral metal accumulation can decrease both pollen and ovule viability due to developmental abnormalities in anthers and ovaries (Yousefi *et al.* 2011*a*, *b*). While we show that Ni is incorporated into pistils, anthers, pollen and nectar, specifically, further work elucidating the effects of floral Ni accumulation on ovule and pollen viability and reproductive success in natural populations will provide necessary information towards understanding the adaptive value of both metal exclusion and metal accumulation. For example, pollen germination for some plant species known to accumulate high concentrations of the metalloid selenium is actually improved by increasing concentrations of selenium in pistils (Prins *et al.* 2011). However, the effects of floral metal accumulation on plant fitness for serpentine species are unknown, though experiments testing these effects are currently underway (G.A.M. and T.L.A., unpubl. res.).

Current data (DeHart *et al.* in review; this study) support the idea that serpentine endemics possess adaptations to elevated metal concentrations in serpentine soils (i.e. reduced uptake and translocation to leaves, reproductive organs and rewards) that non-endemics lack. These results suggest that non-endemic species may be at a fitness disadvantage compared with endemics when growing on serpentine soils. For example, in a series of experiments with *Mimulus guttatus* (Phrymaceae), Searcy and Mulcahy (1985) and Searcy and Macnair (1990) suggested that copper in the pistils of plants could act as a selective filter since seed production was reduced when pollen donors were not adapted to copper-rich soils. Floral metal accumulation may therefore produce a prezygotic isolating mechanism in non-endemic species compared with endemics by decreasing plant fitness when maternal and paternal plants are growing in different soil environments (i.e. serpentine and non-serpentine). In this way, floral metal accumulation may act as a reproductive barrier that favours reproduction between plants growing in similar soil environments, selecting against species that have serpentine and non-serpentine populations in close proximity to each other. Therefore, understanding metal accumulation into flowers and floral rewards is vital not only for identifying potential reproductive costs associated with plant growth on metal-rich soils, such as serpentine, but also for explaining patterns of species distributions, reproductive isolation and plant endemism.

Metal accumulation by plants can be influenced by many factors, including phylogeny (Broadley *et al.* 2001) and maternal effects (Macnair 2002). Therefore, it must be acknowledged that many of the species used in this study, including all of the species in the endemic and indicator categories, belong to the same genus within the Brassicaceae, *Streptanthus*. However, the main comparisons of this study involved endemics vs. both indifferent and indicator species, with the latter group including members of several genera spread across multiple Tribes (*Boechera*: Boechereae; *Erysimum*: Erysimeae; *Hirschfeldia*: Brassiceae; *Streptanthus*: Schizopetaleae; Al-Shehbaz 2010). Therefore, additional work comparing heavy metal accumulation across vegetative and reproductive organs of endemic and non-endemic plants from a variety of plant families (e.g. Asteraceae, Caryophyllaceae, Phrymaceae), and thus taking phylogenetic relationships into account regarding variation in metal accumulation, will contribute towards a more general understanding of edaphic endemism. Though not incorporated in the present study, the application of phylogenetically independent contrasts with paired endemic and non-endemic taxa (e.g. Cunningham *et al.* 1999) would be particularly informative when comparing Ni accumulation across levels of serpentine affinity. Furthermore, because seeds from each taxon were bulk-collected from a single population per species, intra-population level variation, if it exists (e.g. Macnair 2002), would be confounded with species. Our conclusions, however, are robust across affinity groups as each group includes two or more species. In addition, it is unlikely that maternal environmental effects influenced our findings as tissues were only collected from adult plants, and maternal affects generally manifest in earlier life stages (i.e. seeds and seedlings) and decrease with plant age (Roach and Wulff 1987; Lopez *et al.* 2003; Donohue 2009). However, phytoavailable Ni concentrations in serpentine soils are well known to vary, both within and across regions containing serpentine soil (Echevarria *et al.* 2006). This variation can lead to ecotypic variation within species, with some populations being adapted to high phytoavailable Ni concentrations, while others are not (O'Dell and Rajakaruna 2011). Thus, future studies incorporating multiple populations will allow for further resolution of genetic and maternal environment effects on plant Ni accumulation, and whether these vary by serpentine affinity.

## Conclusions

Although the current study does not assess differences in plant fitness or competitive ability between endemic and non-endemic plants, our results highlight consistent differences in heavy metal uptake between endemic and non-endemic serpentine species. While edaphic features of serpentine soils are known to influence plant fitness for non-endemic plants, both directly (e.g. Swope and Stein 2012) and indirectly (e.g. Meindl *et al.* 2013), the specific effects of Ni accumulation on plant reproduction for non-hyperaccumulating serpentine species are not fully understood. Nickel tolerance has been identified as a key feature of serpentine soil tolerance (Brady *et al.* 2005; Alexander *et al.* 2007; O'Dell and Rajakaruna 2011) and is generally accomplished through root sequestration or exclusion, though not all serpentine plant species effectively exclude Ni from above-ground tissues (reviewed in Alexander *et al.* 2007; O'Dell and Rajakaruna 2011). Given the known toxicity of Ni for plant growth and reproduction (see citations in Introduction), our study suggests that endemic and non-endemic plants may differ in reproductive potential (e.g. differences in seed production or pollinator visitation) when grown in serpentine soils due to differential Ni uptake and translocation. While our findings suggest that endemic species possess the ability to limit Ni uptake into above-ground tissues, future work assessing the fitness consequences of growth on serpentine soils will provide valuable information towards understanding edaphic endemism. However, studies like ours are necessary prerequisites for determining whether serpentine endemic and non-endemic species differ in reproductive or competitive capabilities (e.g. Imbert *et al.* 2011) due to differences in physiological response to soils.

## Sources of Funding

This research was funded by a Botany In Action Fellowship from the Phipps Conservatory and Botanical Gardens, an Ivy McManus Diversity Fellowship (University of Pittsburgh) and an Andrew Mellon Predoctoral Fellowship (University of Pittsburgh) to G.A.M., NSF (EAR-IF 0948366) to D.J.B., and NSF (DEB 1020523, 1241006) to T.-L.A.

## Contributions by the Authors

G.A.M. and T.-L.A. designed the experiment and wrote the paper, G.A.M. performed the experiment, and G.A.M. and D.J.B. performed all chemical analyses.

## Conflicts of Interest Statement

None declared.
